# Prevalence of Genotypes and Subtypes of *Gardnerella vaginalis* in South African Pregnant Women

**DOI:** 10.1155/2020/3176407

**Published:** 2020-07-02

**Authors:** Kayla Pillay, Silondiwe Nzimande, Meleshni Naicker, Veron Ramsuran, Partson Tinarwo, Nathlee Abbai

**Affiliations:** ^1^School of Clinical Medicine Laboratory, College of Health Sciences, University of KwaZulu-Natal, South Africa; ^2^KwaZulu-Natal Research Innovation and Sequencing Platform (KRISP), School of Laboratory Medicine and Medical Sciences, University of KwaZulu-Natal, Umbilo Road, Durban, Private Bag 7, Congella, 4013, South Africa; ^3^Centre for the AIDS Programme of Research in South Africa (CAPRISA), University of KwaZulu-Natal, Umbilo Road, Durban, Private Bag 7, Congella, 4013, South Africa; ^4^Department of Biostatistics, Nelson R. Mandela School of Medicine, University of KwaZulu-Natal, Durban, South Africa

## Abstract

**Background:**

*Gardnerella vaginalis*, a microorganism highly linked to bacterial vaginosis (BV), is understudied in terms of genotypic heterogeneity in South African populations. This study investigated the prevalence of *G. vaginalis* genotypes in BV-positive, BV-intermediate, and BV-negative South African pregnant women.

**Methods:**

The study population included *n* = 354 pregnant women recruited from a public hospital in Durban, South Africa. The women provided self-collected vaginal swabs for BV diagnosis by Nugent scoring. For the genotyping assays, the *16S rRNA* and *sialidase A* genes from BV-negative, BV-intermediate, and BV-positive samples were amplified with *G. vaginalis*-specific primers. The*16S rRNA* amplicon was digested with *TaqI* to generate genotyping profiles, and subtypes were determined by correlating *BamHI* and *HindIII* digestion profiles. Phylogenetic analysis was performed on the *16S rRNA* and *sialidase A* sequences. The data analysis was performed with R Statistical Computing software, version 3.6.2.

**Results:**

Two different genotypes, GT1 and GT2, were detected. The most prevalent genotype was GT1. Four subtypes (1, 2B, 2AB, and 2C) were shown to be present. The most prevalent subtype was 2B, followed by subtypes 1, 2C, and 2AB. The phylogenetic analysis of the *16S rRNA* showed the presence of 5 clusters. The tree displayed clusters which contained sequences from the same BV group with different genotypes and subtypes. Clusters with sequences from across the BV groups carrying the same genotype and subtype were present. Diversity of the *sialidase A* across BV groups and genotypes was observed. Finally, the study did not find a significant association (*p* > 0.05) between reported symptoms of abnormal vaginal discharge and genotype harboured.

**Conclusion:**

This study provided the first report on the diversity of *G. vaginalis* in South African pregnant women. Diversity assessments of *G. vaginalis* with respect to genotypes and virulence factors may aid in a greater understanding of the pathogenesis of this microorganism.

## 1. Background

Bacterial vaginosis (BV) is an imbalance of the vaginal microenvironment [1]. The condition is characterized by a lower abundance of “healthy” lactobacilli and overgrowth of diverse anaerobic bacteria such as *Gardnerella*, *Atopobium*, *Mobiluncus*, *Prevotella*, *Bacteroides*, *Anaerococcus*, *Peptostreptococcus*, *Sneathia*, and *Leptotrichia* and members of the class *Clostridia* [2]. Bacterial vaginosis has been associated with preterm birth and poor perinatal outcomes [3]. A strong association between BV and sexually transmitted infections (STIs) has also been reported [4].

*Gardnerella vaginalis* is found in most women with vaginosis and has been reported to be the main cause of clinical signs and symptoms used to diagnose BV [2, 5]. *G. vaginalis* was originally discovered by Leopold [6] who described this microorganism as a “*Haemophilus*-like” species associated with prostatitis and cervicitis. *G. vaginalis* has the necessary virulence factors including production of sialidase which aids in the adherence to the host vaginal epithelium in order to compete with normal vaginal flora for dominance [7]. Sialidase produced by *G*. *vaginalis* degrades mucosal sialoglycans, believed to be important in BV [8]. Additionally, sialidase produced by some strains of *G. vaginalis* has been shown to interfere with host immune modulation resulting in adverse pregnancy outcomes [9].

For over three decades, researchers have been conducting extensive bacterial typing assays, in order to identify different virulence traits among *Gardnerella* spp. [10]. Phenotypic assays have been used to assess the diversity of *Gardnerella* spp. based on their biochemical properties such as production of b-galactosidase, lipase, and hippurate hydrolysis. However, the early typing assays had failed to reveal the diversity of *G*. *vaginalis*^5^ [8],. The genetic heterogeneity of *G. vaginalis* species has been determined using molecular approaches, such as Amplified Ribosomal DNA restriction analysis (ARDRA) [11]. ARDRA is a simple, fast, and reproducible method for microbial molecular epidemiology and taxonomy [12]. The ARDRA genotyping approach developed by Balashov and coworkers was shown to be less error-prone [13]. In the study by Ingianni et al. [14], the ARDRA method allowed for *G. vaginalis* to be separated into at least 4 genotypes.

Despite the availability of useful genotyping techniques for *G. vaginalis*, it has been documented that there is limited data on the prevalence of *G. vaginalis* genotypes from across the globe [15]. This study investigated the diversity of *G. vaginalis* from noncultured vaginal swabs obtained from pregnant women by ARDRA.

Past studies have described a link with sialidase production and particular *G. vaginalis* ARDRA genotypes [9, 15]. A recent study by our research group had found high copy numbers of the *sialidase A* gene across BV-intermediate and BV-positive women and in women with and without abnormal vaginal discharge (unpublished). However, the association between sialidase and *G. vaginalis* ARDRA genotypes was not performed. This current study will attempt to fill this gap in evidence. In addition, no clear association between BV and any of the ARDRA genotypes has been reported [11]. Through this study, the distribution of *G. vaginalis* ARDRA genotypes linked to BV status and clinical symptoms of BV such as abnormal vaginal discharge will be determined.

## 2. Methodology

### 2.1. Ethics Approval

The study was approved by the Biomedical Research Ethics Committee (BREC) of the University of KwaZulu-Natal (BREC/00000093/2019).

### 2.2. Study Population

A population of pregnant women was recruited from the King Edward VIII hospital in Durban, South Africa. The study population was recruited from October 2017 to April 2018. The enrolled women (*n* = 354) provided self-collected vaginal swabs after receiving instructions from the study staff on the method of sample collection. Samples were collected from women from gestational age 12 weeks to 37 weeks. The women were classified as BV-negative, BV-intermediate, and BV-positive using the Nugent scoring criteria on gram-stained vaginal smears. A 100% in-house quality control check on the gram-stained slides was performed. The study was conducted at the School of Clinical Medicine Research Laboratory at the Nelson R. Mandela School of Medicine, University of KwaZulu-Natal.

### 2.3. Laboratory Procedures

#### 2.3.1. Nugent Scoring for Grading of Vaginal Smears

Smears were prepared from vaginal swabs and rolled onto glass slides. The slides were gram-stained and examined under the oil immersion objective. Each slide was then graded as per the standardized quantitative morphological classification method developed by Nugent et al. [16].

#### 2.3.2. DNA Extraction

DNA was extracted from the vaginal swabs using a commercially available kit, PureLink Microbiome DNA purification kit (ThermoFisher Scientific, Massachusetts, United States), according to the manufacturer's instructions.

#### 2.3.3. Amplification of the *16S Ribosomal RNA* of *G. vaginalis*

The *16S rRNA* gene specific to *G. vaginalis* was amplified using primers: forward: 5′-TTCGATTCTGGCTCAGG and reverse: 5′-CCATCCC AAAAGGGTTAGGC. The primers were synthesized based on their published sequences described by Pleckaityte et al. [15]. The PCR was performed in a 50 *μ*L final volume and comprised 0.2 *μ*M of each primer, 30 ng of genomic DNA, and 1.5 U of High-Fidelity PCR enzyme mix (ThermoFisher Scientific, Massachusetts, United States). The reaction mixture was subjected to 28 cycles of denaturation at 94°C for 30 seconds, primer annealing at 52°C for 45 seconds, and extension at 72°C for 1 minute 25 seconds. PCR conditions were as per Pleckaityte et al. [15]. All PCR reactions were performed using a T100 thermocycler (BioRad, California, United States). The PCR products were separated on a 1% agarose gel and viewed under a UV transilluminator (Gene Genius, SYNGENE).

#### 2.3.4. Sequence Analysis of the *16S Ribosomal RNA*

To confirm the identity of the PCR amplicons prior to genotyping, the amplicons were sequenced using the Sanger method [17] at Inqaba Biotechnological Industries in Pretoria, South Africa. The amplicons were sequenced using an ABI3500XL genetic analyser, and the raw sequence data was edited using Chromas software V2.6.5 (Technelysium, Queensland, Australia). The edited forward and reverse sequences were aligned using the DNAMAN software (Lynnon Biosoft, California, United States), and the identity of the edited sequences was confirmed using the National Center for Biotechnology Information (NCBI) Basic Local Alignment Search Tool (BLAST).

#### 2.3.5. Amplified Ribosomal DNA Restriction Analysis (ARDRA)

The PCR products were subjected to restriction analysis with *TaqI*, *BamHI*, and *HindIII* (New England Biolabs, Massachusetts, United States). Digestion with *TaqI* was performed at 65°C for 3 hours, and digestion with *BamHI* and *HindIII* was performed at 37°C for 4 hours. Restriction products were analysed on a 1.5% agarose gel (SeaKem LE Agarose-Lonza, Maine, United States) electrophoresed in Tris-borate-EDTA (TBE) buffer.

#### 2.3.6. Phylogenetic Analysis of Genotypes

A phylogenetic tree was then constructed from the *16S rRNA* sequence data using the Molecular Evolutionary Genetics Analysis (MEGA) version 10 software (Arizona, United States). A bootstrap consensus tree inferred from 100 replicates using the Neighbour Joining method was generated [18].

#### 2.3.7. Detection of the *Sialidase A* Gene from *G. vaginalis*

The presence of the *sialidase A* gene was detected using the following specific primers: forward: 5′-GACGACGGCGAATGGCACGA-3′ and reverse: 5′-TACAAGCGGCTTTACTCTTG-3′. The primers were synthesized based on their published sequences described by Pleckaityte et al. [15]. The PCR conditions were as follows: an initial denaturation at 95°C for 10 minutes was followed by 40 cycles of denaturation at 95°C for 30 seconds, annealing was performed at 58°C for 1 minute and extension at 72°C for 2 minutes, and this was followed by final extension at 72°C for 7 minutes. All PCR reactions were performed using a T100 thermocycler (BioRad, California, United States). PCR products were separated on a 1% agarose gel and viewed under a UV transilluminator (Gene Genius, SYNGENE).

#### 2.3.8. Sequence Analysis of *Sialidase A*

Amplicons generated using the *sialidase A*-specific primers were sequenced using the Sanger method as previously described. The sequence data generated for the *sialidase A* gene was compared across the genotypes.

#### 2.3.9. Data Analysis

The data analysis was performed in R Statistical Computing software, version 3.6.2. To assess the association between the symptoms and the BV status for each genotype, the Chi-squared goodness of fit test for one sample was used. The results were also presented as component bar charts.

## 3. Results

### 3.1. BV Diagnosis

Of the 354 samples analysed, 124 were BV-positive, 37 were BV-intermediate, and 113 were BV-negative. The remaining slides (100) were unreadable due to poor quality of the slide (inadequate sample material on slide). We randomly selected 50 BV-negative, 37 BV-intermediate, and 50 BV-positive specimens for the genotypic analysis. A total of 137 samples were analysed.

### 3.2. Amplification of the *16S Ribosomal RNA* of *G. vaginalis*

The 1300 bp fragment corresponding to the *16S rRNA* of *G. vaginalis* was only amplified in 37 of the 137 samples analysed (27.2%) (data not shown). The *16S rRNA* gene was not detected in any of the BV-negative samples (0/50). A BV-negative group was therefore not included in any further analysis. From the 50 BV-positive samples based on Nugent scoring, only 23 samples produced the 1300 bp product. In addition, 14/37 BV-intermediate samples were amplifiable. Attempts to generate amplicons for the unsuccessful samples were investigated such as increasing the concentration of template DNA and adjusting primer and amplification conditions. All attempts were unsuccessful. The possibility of sample inhibitors affecting the PCR reactions or failed DNA extractions was ruled out since the same DNA samples were amplifiable for other genes not included in this study. A set of 37 samples were used for further analysis. The DNA sequencing hits of the *16S rRNA* showed identity (97%) to *G. vaginalis* strain GS10234 (MH898659.1) and *G. vaginalis* strain N153 (98%) (JQ354973.1).

### 3.3. Genotyping Analysis

The distribution of the genotypes based on *TaqI* digestion for the 37 specimens analysed is shown in Table 1. The subtypes of the genotypes which were determined by combining the banding profiles of *BamHI* and *HindIII* digestions are also presented in Table 1.

#### 3.3.1. Genotypes Based on *TaqI* Digestion

Restriction digestion with *TaqI* revealed the presence of two different genotypes, i.e., GT1 and GT2. GT1 was carried by 20/37 specimens (54%), followed by GT2 which was present in 9/37 specimens (24%). Of the 37 specimens analysed, 7 specimens were not ascribed genotypes. Two of the specimens from the BV-positive sample group produced a banding profile (i.e., a single band at 500 bp) that was not described in previously published studies. One specimen from the BV-intermediate group produced a very faint profile which was difficult to interpret. The remaining 3 specimens did not produce any bands; the gel lanes appeared blank for those samples. These specimens were across both BV status groups.

**Figure 1:**
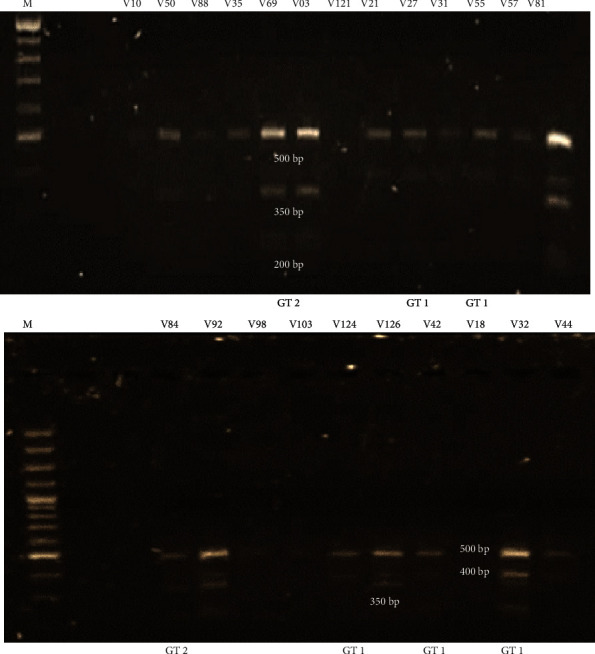
Profiles of BV-positive samples digested with *TaqI*. Lane M: 100 bp DNA molecular ladder (ThermoFisher Scientific). Genotypes 1 (100 bp, 350 bp, 400 bp, and 500 bp banding patterns) and 2 (100 bp, 200 bp, 350 bp, and 500 bp banding patterns) were distributed across BV-positive samples.

Within the BV-positive sample group, 13/23 specimens carried GT1 (57%) and 6 of the 23 specimens (26%) carried GT2. Two specimens produced a differing banding profile (9%), and 2 specimens did not produce any bands (9%) (Figure 1).

**Figure 2:**
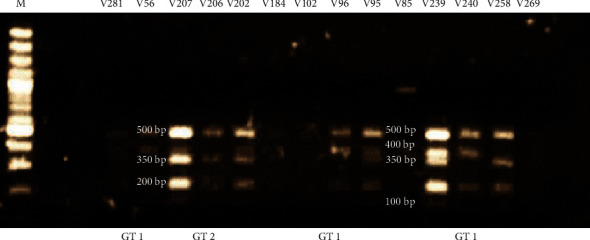
Profiles of BV-intermediate samples digested with *TaqI*. Lane M: 100 bp DNA molecular ladder (ThermoFisher Scientific). Genotypes 1 (100 bp, 350 bp, 400 bp, and 500 bp banding patterns) and 2 (100 bp, 200 bp, 350 bp, and 500 bp banding patterns) were distributed across BV-intermediate samples.

A similar profile was observed for the BV-intermediate sample group; a larger number of specimens carried GT1 (7/14, 50%), and 3 out of 14 samples carried GT2 (21%). Three samples did not produce visible bands (21%) (Figure 2).

#### 3.3.2. Subtypes Based on *BamHI* and *HindIII* Digestions

**Figure 3:**
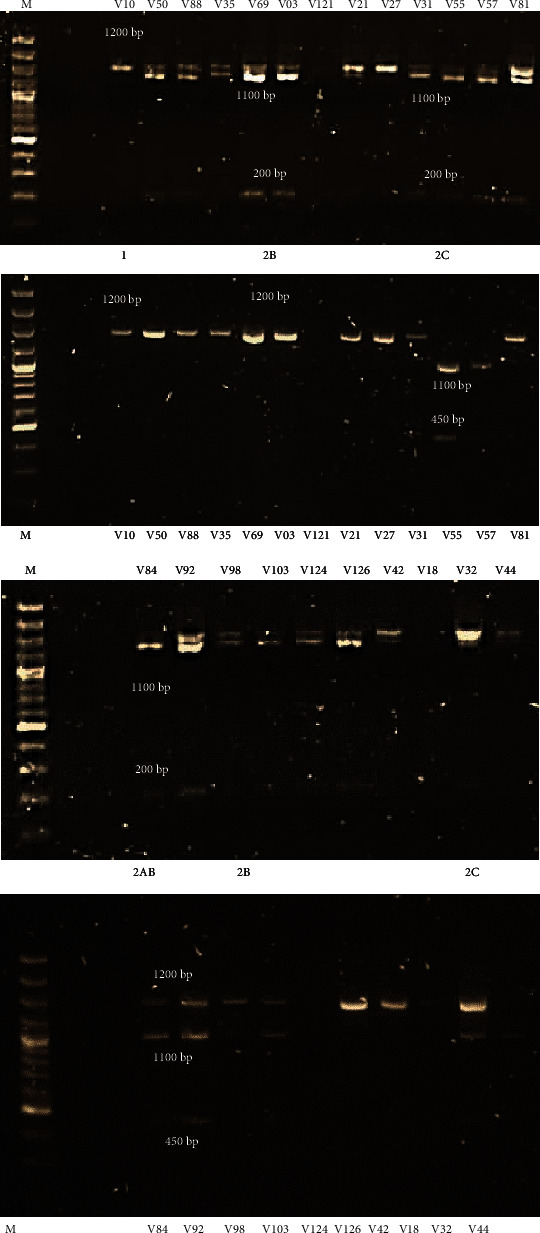
Subtypes assigned to BV-positive samples based on *BamHI* and *HindIII* digestion profiles. Lane M: 100 bp DNA molecular ladder (ThermoFisher Scientific). Subtypes 1 (1200 bp band for *BamHI* and *HindIII* digestions), 2B (200 bp and 1100 bp for *BamHI* digestion and 1200 bp for *HindIII* digestion), 2AB (200 bp and 1100 bp for *BamHI* digestion and 450 bp, 1000 bp, and 1200 bp for *HindIII* digestion), and 2C (200 bp and 1100 bp for *BamHI* digestion and 450 bp and 1000 bp for *HindIII* digestion) were distributed across the BV-positive samples.

Within the BV-positive sample group, all 4 subtypes were observed. Subtype 2B was highly prevalent with 11/23 (48%) specimens harbouring this subtype followed by the mixed 2AB subtype (4/23, 17%), subtype 2C (3/23, 13%), and subtype 1 (2/23, 9%) (Figure 3). In the sample group that carried GT1 (*n* = 13), 5 specimens harboured subtype 2B (39%), 3 with subtype 2C (23%), 2 with subtype 2AB (15%), and 2 with subtype 1 (15%) (Table 1). Subtype 2B was highly prevalent in the sample group carrying GT2 (5/6, 83%), followed by subtype 2AB (1/6, 17%). Subtypes 1 and 2C were not present in this genotypic group (Table 1).

**Figure 4:**
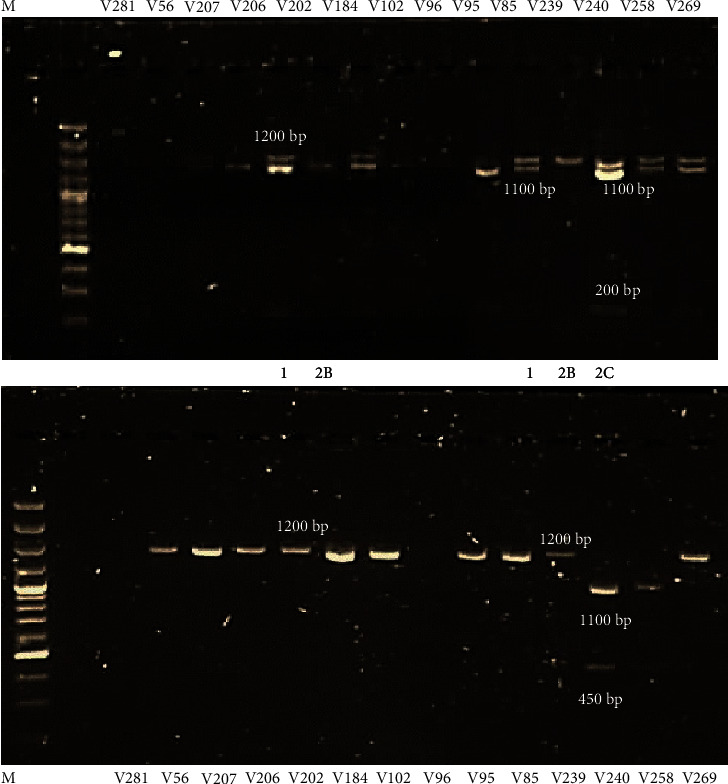
Subtypes assigned to BV-intermediate samples based on *BamHI* and *HindIII* digestion profiles. Lane M: 100 bp DNA molecular ladder (ThermoFisher Scientific). Subtypes 1 (1200 bp band for *BamHI* and *HindIII* digestions), 2B (200 bp and 1100 bp for *BamHI* digestion and 1200 bp for *HindIII* digestion), and 2C (200 bp and 1100 bp for *BamHI* digestion and 450 bp and 1000 bp for *HindIII* digestion) were distributed across the BV-intermediate samples.

Within the BV-intermediate sample group, 3 subtypes were observed (subtypes, 1, 2B, and 2C) (Figure 4). The most prevalent subtype in this group was subtype 1 (9/14, 64%) followed by subtype 2B (4/9, 44%) and subtype 2C (1/9, 11%). Subtypes 1 (3/7, 43%) and 2B (3/7, 43%) were most prevalent in GT1 specimens. One specimen in this genotypic group carried subtype 2C (7%) (Table 1). Subtype 1 was also shown to be most prevalent in the GT2 specimens (2/3, 67%) followed by subtype 2B (1/3, 33%). Subtype 2C was not present in this genotypic group (Table 1).

### 3.4. Phylogenetic Analysis of *16S rRNA* Genotypes and Subtypes

**Figure 5:**
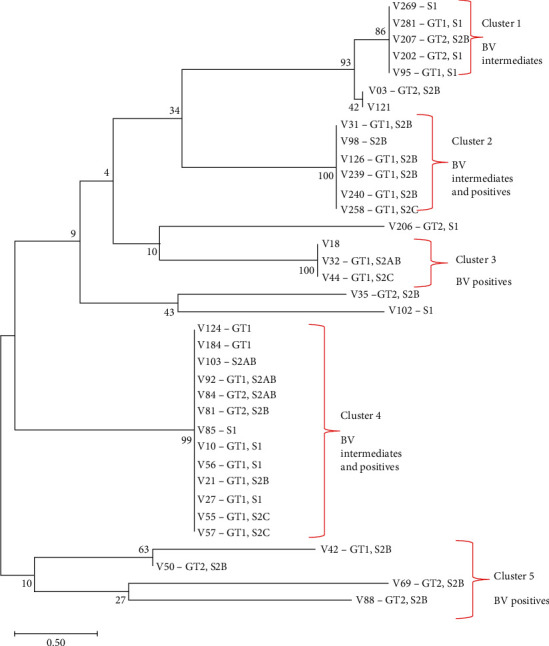
Phylogenetic analysis according to distribution of genotypes. The tree was constructed using the Neighbour Joining method. The optimal tree with the sum of branch length = 15.35302543 is shown. The percentage of replicate trees in which the associated taxa clustered together in the bootstrap test (100 replicates) is shown next to the branches. The tree is drawn to scale, with branch lengths in the same units as those of the evolutionary distances used to infer the phylogenetic tree. The evolutionary distances were computed using the Maximum Composite Likelihood method and are in the units of the number of base substitutions per site. Evolutionary analyses were conducted in MEGA X [18].

The phylogenetic tree revealed the presence of 5 sequence clusters (Figure 5). The tree displayed clusters which contained groups of specimens from a particular BV group (clusters 1, 3, and 5). Within these same BV groups, there were however differences noted for either the genotypes assigned and/or subtypes present. Additionally, there were clusters which contained specimens from across both BV groups such as clusters 2 and 4. Despite the heterogeneity with respect to the BV group, cluster 2 contained specimens of the same genotype (GT1) with the majority carrying the same subtype (S2B). Cluster 4 on the other hand contained specimens of the same genotype (GT1) with a combination of all 4 subtypes (S1, S2B, S2AB, and S2C).

### 3.5. Symptoms Associated with BV across Genotypes and BV States

**Figure 6:**
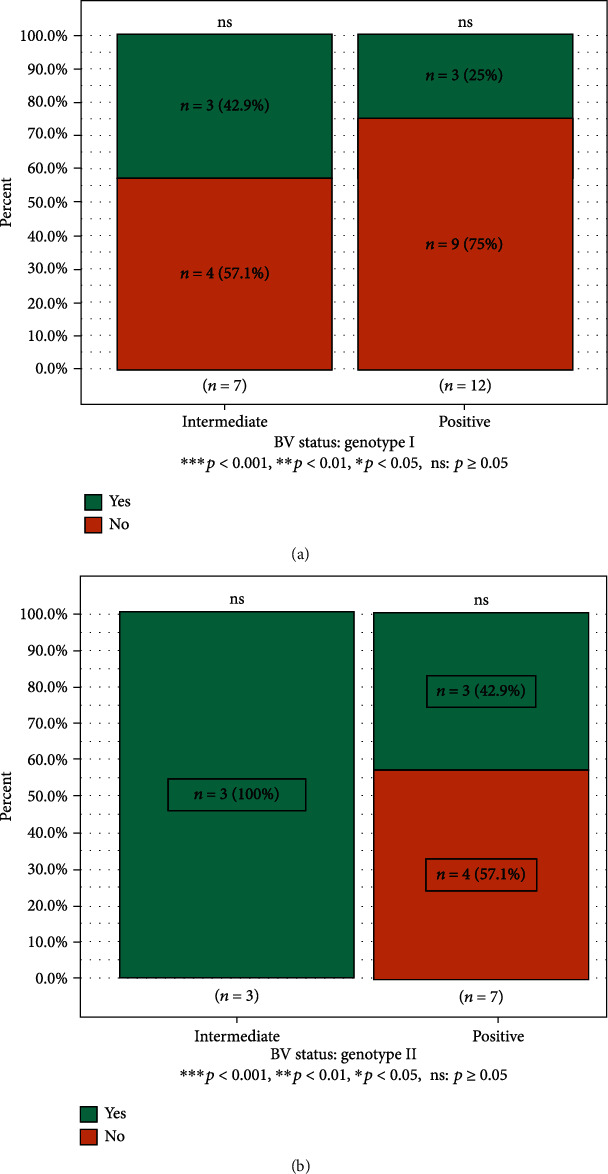
Symptoms of bacterial vaginosis across the intermediate and positive women in relation to genotypes. ns = not significant.

The median age (Q1-Q3) of the women in the BV-intermediate group was 26.5 (21.3-28.8), and the median age (Q1-Q3) of the women in the BV-positive group was 26.0 (24.0-30.5). Among the BV-intermediate and BV-positive groups, a higher percentage of the women did not present with symptoms of abnormal vaginal discharge (i.e., were asymptomatic). For BV-intermediate and BV-positive women harbouring GT1, there was no significant difference in women who reported abnormal vaginal discharge when compared to women who did not report the discharge (*p* > 0.05) (Figure 6(a)).

Similarly, for the women harbouring GT2, there was no significant difference in the BV-positive women who reported abnormal vaginal discharge when compared to women who did not report discharge (*p* > 0.05). All BV-intermediate women with GT2 reported no symptoms of abnormal vaginal discharge (Figure 6(b)).

### 3.6. Sequence Diversity of *Sialidase A* Linked to the *G. vaginalis* Genotypes

**Figure 7:**
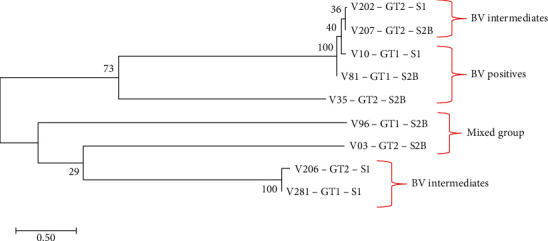
Phylogenetic analysis of *sialidase A* in relation to genotype. The tree was constructed using the Neighbour Joining method. The evolutionary history was inferred using the Neighbour Joining method. The optimal tree with the sum of branch length = 0.18320599 is shown. The percentage of replicate trees in which the associated taxa clustered together in the bootstrap test (100 replicates) is shown next to the branches. The tree is drawn to scale, with branch lengths in the same units as those of the evolutionary distances used to infer the phylogenetic tree. The evolutionary distances were computed using evolutionary analyses which were conducted in MEGA X [18].

The *sialidase A* gene was detected in both BV-intermediate and BV-positive women. A subset of *sialidase A*-positive amplicons from samples representing the different genotypes across the BV groups was sequenced and analysed. Four *sialidase A* sequence clusters were observed (Figure 7). Cluster 1 contained sequences from BV-intermediate samples harbouring the same genotype (GT2) but different subtypes. Cluster 2 contained sequences from BV-positive samples harbouring the same genotype (GT1) but different subtypes. Cluster 3 was a heterogeneous group containing sequences from both BV-intermediate and BV-positive groups harbouring different genotypes but the same subtype. Lastly, cluster 4 contained sequences from BV-intermediate samples harbouring the different genotypes but the same subtype. Overall, diversity of the *sialidase A* in terms of BV group and genotypes harboured was observed.

## 4. Discussion

*Gardnerella vaginalis* is one of the most frequently isolated microorganisms from women who present with symptoms of BV [19]. High microbial loads of *G. vaginalis* in the vaginal tract have been linked to reproductive health issues such as infertility and preterm labour [20]. The pathogenesis of *G. vaginalis* in the vaginal tract is not completely understood since this microorganism has been shown to be present across the BV groups (BV-negative, BV-intermediate, and BV-positive). Differentiation of *G. vaginalis* strains and subgroups according to sequence variations in *16S rRNA* and the *cpn60* genes has been made possible using molecular biology approaches [13].

In this study, the diversity of the *G. vaginalis 16S rRNA* was analysed across BV-intermediate and BV-positive pregnant women who were diagnosed by the Nugent method. A BV-negative group was not included in the diversity analysis since none of the BV-negative samples produced a positive PCR amplicon for the *16S rRNA* specific for *G. vaginalis*. However, the presence of *Lactobacillus crispatus* was shown to be present in the negative specimens eliminating the possibility of a failed DNA extraction or PCR amplification for the negative specimens (data not shown). Our failure to amplify the *G. vaginalis 16S rRNA* in the negative samples differs from previously published works which had shown the presence of *G. vaginalis* in BV-negative specimens based on PCR detection of the *16S rRNA* gene [13, 21]. However, a fairly recent study conducted by our research group was able to detect *G. vaginalis* in our BV-negative pregnant cohort using the highly sensitive Droplet Digital PCR (ddPCR) System (*submitted for publication*). This leads to the assumption that ARDRA may not be a very sensitive method for detecting *G. vaginalis* directly from clinical samples in women classified as BV-negative. Our assumption is validated by an earlier study conducted by Verhelst et al. [22] which used ARDRA in order to assess the diversity of the vaginal microbiome. In that study, ARDRA failed to identify *G. vaginalis* in women who were classified as BV-negative; however, *G. vaginalis* was detected in women who were BV-positive. Despite the suggested limitation, ARDRAhas been useful in identifying different *G. vaginalis* genotypes [9, 14, 23].

Based on the ARDRA technique used in this study, restriction digestion with *TaqI* revealed the presence of two different genotypes, i.e., GT1 and GT2. Similarly, a study by Pleckaityte et al. [15] reported on the presence of GT1 and GT2 in a population of women with BV in Lithuania. However, the Lithuanian study was unable to detect specific subtypes associated with GT1. All the GT1 sequences in their population of women were identical. However, the present study observed different subtypes associated with GT1. This suggests that a level of diversity does exist between *G. vaginalis* present in different geographical locations as well as across different population groups (pregnant versus nonpregnant). Additionally, the present study observed a difference in the prevalence of the different subtypes across BV-intermediate and BV-positive women. Among the BV-positive women, the most prevalent subtype was 2B whereas in the BV-intermediate women, the most prevalent subtype was subtype 1, thereby hypothesising a level of genetic differences across BV-intermediate and BV-positive women. Our hypothesis was confirmed by the phylogenetic analysis which showed the presence of 5 sequence clusters on the tree indicating genetic differences across the sequences.

The study further investigated the link between genotypes and clinical symptoms of abnormal vaginal discharge. Among the BV-intermediate and BV-positive groups, a higher percentage of the women did not present with symptoms of abnormal vaginal discharge (i.e., were asymptomatic). This study found no significant association between genotypes harboured and symptoms of abnormal vaginal discharge. However, a study conducted by Santiago et al. [9] showed GT2 to be the most prevalent genotype associated with symptomatic BV.

This study also investigated the diversity of the *sialidase A* gene, the virulence factor of *G. vaginalis* in relation to genotypes. In this study, there was a correlation between the DNA sequences of the *sialidase A* gene and the respective genotypes. Two of the four sequence clusters contained samples of the same genotype. A clear link between genotype and sialidase production was previously reported by Santiago et al. [9]. However, the present study cannot be directly compared to that of Santiago et al. [9] since the present study detected the *sialidase A* gene directly from noncultured vaginal swabs whereas Santiago et al. [9] evaluated pure cultures for sialidase activity and diversity. The present study also showed that the diversity of the *sialidase A* gene was based on the BV group. BV-intermediate and BV-positive sequences formed distinct clusters on the phylogenetic tree indicating a level of diversity of *sialidase A* gene across the two BV groups. Previous studies on the diversity of *sialidase A* gene were not investigated in women with intermediate BV. The current study now provides additional data on the diversity of the *sialidase A* gene for this BV group.

The limitations of the study are as follows: the sample size used for the analysis was small. However, despite the small sample size, the study was able to provide data on the prevalent genotypes and subtypes of *G. vaginalis* in South African pregnant women across BV-intermediate and BV-positive groups, an area of research which has not been previously investigated in our setting. The study lacked a control group of nonpregnant women which would have been useful to draw comparisons regarding the distribution of the genotypes. The study did not attempt to culture *G. vaginalis* from the vaginal swabs and therefore did not perform the genotyping assays on pure cultures that would have enabled direct comparisons with previously published studies. However, from the noncultured clinical specimens, the diversity assessments performed still provided substantial evidence. Lastly, due to the cross-sectional nature of the study, we did not associate the genotypes with pregnancy outcomes and acquisition of other infections such as HIV and genital infections. All the limitations described here will be addressed in a study that is planned for commencement in 2021.

## 5. Conclusion

This study provides the first report on the most prevalent genotypes and subtypes of *G. vaginalis* across BV-intermediate and BV-positive South African pregnant women. Restriction analysis revealed the presence of two different genotypes, i.e., GT1 and GT2, as well as four subtypes (1, 2B, 2AB, and 2C) circulating in our population. In addition, diversity across the BV groups, genotypes, and subtypes for *sialidase A* was evident in this study. The observed diversity can be used as a foundation for future studies which are aimed at understanding the pathogenesis of *G. vaginalis* across BV groups in women from different populations.

## Figures and Tables

**Table 1:** Genotypes identified after digestion with *TaqI* across BV-intermediate and BV-positive women. Subtypes identified after digestion with *BamHI* and *HindIII*.

Sample name	*TaqI* fragment sizes	Genotype	*BamHI* fragment sizes	*HindIII* fragment sizes	*Subtype*
*BV positives*					
V003	100 bp, 200 bp, 350 bp, 500 bp	2	200 bp, 1100 bp	1200 bp	2B
V010	350 bp, 400 bp	1	1200 bp	1200 bp	1
V018	Bands not visible	—	Bands not visible	Bands not visible	—
V021	100 bp, 350 bp, 400 bp, 500 bp	1	200 bp, 1100 bp	1200 bp	2B
V027	100 bp, 350 bp, 400 bp, 500 bp	1	1200 bp	1200 bp	1
V031	100 bp, 350 bp, 400 bp, 500 bp	1	200 bp, 1100 bp	1200 bp	2B
V032	250 bp, 350 bp, 400 bp, 500 bp	1	200 bp, 1100 bp	450 bp, 1000 bp, 1200 bp	2AB
V035	350 bp, 500 bp	2	200 bp, 1100 bp	1200 bp	2B
V042	400 bp, 500 bp	1	200 bp, 1100 bp	1200 bp	2B
V044	400 bp, 500 bp	1	200 bp, 1100 bp	450 bp, 1000 bp	2C
V050	100 bp, 200 bp, 350 bp, 500 bp	2	200 bp, 1100 bp	1200 bp	2B
V055	100 bp, 350 bp, 400 bp, 500 bp	1	200 bp, 1100 bp	450 bp, 1000 bp	2C
V057	100 bp, 350 bp, 400 bp, 500 bp	1	200 bp, 1100 bp	450 bp, 1000 bp	2C
V069	100 bp, 200 bp, 350 bp, 500 bp	2	200 bp, 1100 bp	1200 bp	2B
V081	100 bp, 350 bp, 400 bp, 500 bp	1	200 bp, 1100 bp	1200 bp	2B
V084	100 bp, 200 bp, 350 bp, 500 bp	2	200 bp, 1100 bp	450 bp, 1000 bp, 1200 bp	2AB
V088	350 bp, 500 bp	2	200 bp, 1100 bp	1200 bp	2B
V092	100 bp, 350 bp, 400 bp, 500 bp	1	200 bp, 1100 bp	450 bp, 1000 bp, 1200 bp	2AB
V098	500 bp	Different patterns	200 bp, 1100 bp	1200 bp	2B
V103	500 bp	Different patterns	200 bp, 1100 bp	450 bp, 1000 bp 1200 bp	2AB
V121	Bands not visible	—	Bands not visible	Bands not visible	—
V124	400 bp, 500 bp	1	200 bp, 1100 bp	Bands not visible	—
V126	100 bp, 350 bp, 400 bp, 500 bp	1	200 bp, 1100 bp	1200 bp	2B
*BV intermediates*					
V056	250 bp, 400 bp, 500 bp	1	1200 bp	1200 bp	1
V085	Undigested DNA	—	1200 bp	1200 bp	1
V095	250 bp, 350 bp, 400 bp, 500 bp	1	200 bp, 1100 bp	1200 bp	1
V096	250 bp, 400 bp, 500 bp	1	200 bp, 1100 bp	1200 bp	2B
V102	Very faint pattern	—	1200 bp	1200 bp	1
V184	No band visible	—	1200 bp	1200 bp	1
V202	250 bp, 350 bp, 500 bp	2	200 bp, 1100 bp	1200 bp	1
V206	250 bp, 350 bp, 500 bp	2	1200 bp	1200 bp	1
V207	250 bp, 350 bp, 500 bp	2	200 bp, 1100 bp	1200 bp	2B
V239	250 bp, 350 bp, 400 bp, 500 bp	1	200 bp, 1100 bp	400 bp, 1000 bp	2B
V240	250 bp, 400 bp, 500 bp	1	200 bp, 1100 bp	1200 bp	2B
V258	250 bp, 400 bp, 500 bp	1	200 bp, 1100 bp	450 bp, 1000 bp	2C
V269	No bands visible	—	1200 bp	1200 bp	1
V281	250 bp, 400 bp, 500 bp	1	1200 bp	1200 bp	1

## Data Availability

The data will be made available upon request.
